# Offline and Online Gambling in a Swiss Emerging-Adult Male Population

**DOI:** 10.1007/s10899-022-10106-w

**Published:** 2022-02-08

**Authors:** Alexander Tomei, Gordana Petrovic, Olivier Simon

**Affiliations:** Centre for Excessive Gambling, Addiction Medicine, Lausanne University Hospital and University of Lausanne, Rue du Bugnon 23, CH-1011 Lausanne, Switzerland

**Keywords:** Gambling, Online gambling, Males, Emerging adults, Switzerland

## Abstract

Faced with the rapid expansion of the online gambling offer, a growing number of jurisdictions around the world are developing legislation to regulate online gambling. The new Swiss Act on gambling extends legalization of online gambling from already authorized lottery-type to casino-type gambling. In this context, the present study examines offline and online gambling behaviors in emerging-adult males as a particularly at-risk population for gambling addiction. A sample of 1,869 young males completed a questionnaire assessing offline and online gambling behaviors as well as severity of problem gambling. Results show that 62.1% of the respondents were lifetime gamblers while 46.7% were past-year gamblers. Among the latter, 76.4% had gambled only within offline venues, 21.4% had gambled at both offline and online sites, and 2.2% had gambled online only. Furthermore, 17.6% of past-year gamblers were at moderate-risk of developing problem gambling whilst 3.6% were problem gamblers. Association analysis showed that, compared to non-problem gamblers, at-risk and problem gamblers played online at higher rates. These findings contribute to the growing literature on youth gambling behaviors and on offline-online transitions. In the context of the growing online gambling supply, the evolution of youth gambling behaviors should be monitored through periodical assessments.

## Introduction

Being young and male are two consistent demographic correlates of gambling frequency and problem gambling behaviors (Williams et al., 2012). Gambling is already present as an established activity amongst adolescents (Calado et al., 2017; Montiel et al., 2021). However, involvement in gambling activities reaches a peak in the years following adolescence (Hollén et al., 2020; Welte et al., 2011). During this period of life, defined as emerging adulthood, the individual explores and seeks new experiences often through risk-taking behaviors, including driving at high speed, unprotected sex and binge drinking (Arnett, 2000). As such, young people are more exposed to the risk of developing an addictive disorder (Sussman & Arnett, 2014). A few studies have examined gambling behaviors in emerging adults by reporting data specific to male participants. According to these studies, male participation in past-year gambling varies between approximately 44% and 69% (Hayatbakhsh et al., 2012; Hollén et al., 2020; Scholes-Balog et al., 2014; Tomei et al., 2015; Wardle & McManus, 2021). Regarding problem gambling, one study has reported that 4.9% of their male sample were problem gamblers (Wardle & McManus, 2021). Another study found that 8.4% of male gamblers met the criteria for problem gambling (Scholes-Balog et al., 2014).

Over the past two decades, the expansion of online gambling has increased public health concerns. Most of the concerns emerge from the dramatic change introduced by online gambling relative to offline gambling in terms of reducing spatial, temporal and monetary constraints. Problem-gambling prevention initiatives are reported to be effective for terrestrial gambling. These include restricting the location of gambling venues, limiting gambling venues’ hours of activity, and restricting access to money (Williams et al., 2012). However, such prevention initiatives do not apply to online gambling. Through mobile devices such as tablets and smartphones, gamblers can access games anywhere, at any time, and for an unlimited duration. In addition, when gambling online, money is exclusively virtual, easily available, and at hand while gambling. Furthermore, online gambling is anonymous and performed in private locations where no form of monitoring exists. Moreover, the structural properties of online games appear similar to those of electronic games, which have been shown to prolong gambling activities at the machine (e.g. Côté et al., 2003; Delfabbro et al., 2005). In fact, online games may be configured to increase the frequency of wages through an increase in the frequency of rewards, and to reduce latency between rewards. These characteristics contribute to the development and maintenance of problem gambling (Griffiths & Auer, 2012).

To date, there is no clear evidence of a direct relationship between the opening of the online gambling market and the prevalence of problem gambling in the general population (Wood et al., 2012). Yet, this relationship has been proven to exist specifically for emerging adults, and particularly among online poker players (Abbott et al., 2014). Moreover, there is mounting evidence that the use of online gambling is associated with problem gambling (Gainsbury, 2015; Williams & Wood, 2007; Wood & Williams, 2011), with online gamblers being more likely to develop problem gambling than offline gamblers. Additionally, such relationships are mainly found among youths (Chóliz, 2016; Hing et al., 2015; Petry & Weinstock, 2007; Potenza et al., 2011; Wijesingha et al., 2017), and among males (McCormack et al., 2014).

To respond to the growth of online gambling, jurisdictions around the world are enacting new laws to regulate online gambling. For its part, in 2019, Switzerland has put into force a new federal Act on gambling (Swiss Confederation, 2017). The new act intends to modernize and harmonize pre-existing law and open the market for online casino gambling. Specific provisions aim to ensure that the level of danger associated with different games is taken into account during licensing. Particularly, games for online use must be specifically designed for accompaniment by protection measures. In addition, only authorized Swiss online-gambling sites are permitted (unauthorized sites are to be blocked), which should serve to limit gambling opportunities whilst providing legal offers and protecting vulnerable consumers. In addition, the new Act and its related ordonnance include provisions for the prohibition of advertising targeting minors and/or advertising likely to mislead the public, particularly over the probability of winning. Finally, electronic lotteries operating outside casinos are subject to a specific age control system. However, the new law does not include provisions to address microtransactions.

In this Swiss public health context, it is important to assess online gambling behaviors in vulnerable populations such as emerging adults. A previous study on emerging adults in Switzerland reported that 56.1% of 18–22 years old males were past-year gamblers and 13.7% had gambled at least once a week (Tomei et al., 2015). Additionally, scratch cards were the most popular game played, and 16% of gamblers had placed bets on the Internet. Furthermore, the study reported that approximately 12% of the sample were at-risk and problem gamblers. However, the Tomei et al. (2015) study was limited in that it examined gambling behaviors as a whole, without a specific investigation into online gambling behaviors. In addition, the study used a small sample. The purpose of the present study is therefore to address these limitations. Particularly, we will examine both offline and online gambling behaviors in a population of emerging-adult males from the French-speaking region of Switzerland. Specifically, the study aims to (a) examine offline and online gambling behaviors separately, (b) assess the severity of problem gambling in this population, and (c) examine the association between gambling behaviors and problem gambling. In addition, it includes a larger sample than that used previously. By fulfilling these goals, the present study provides data for a better understanding of gambling behaviors in emerging adults in Switzerland. Moreover, the data gathered here could be useful as a threshold for subsequent cross-sectional studies to monitor gambling behaviors in this vulnerable population.

## Methods

### Sample

2,203 conscripts from the Swiss Army took part in the study. Approximately 15% of these (n = 334) were excluded from the sample due to age under-representativeness (affecting weighting; n = 210), being female (n = 63), incomplete questionnaires (n = 54), or residing abroad (n = 7). Therefore, the final sample constituted 1869 participants, all males, aged 18–22 years (M = 19.1; Md = 19). Most of the participants were single (95%), 0.5% were married, 4.3% were not married but lived with a partner, and two of them were widowers. About half of the sample (52.3%) were students, 27.7% had a paid professional activity, 15.4% were unemployed, 0.9% were on welfare and around 3.7% were both students and employees or undertook other activities. With regard to education, 3.9% of the respondents did not have any qualifications, 34% had a lower secondary level of education, 23% had an upper secondary level education 32.2% had completed vocational training, 4.7% had completed tertiary level education, and 2.2% had other qualifications.

### Procedure

Data were collected using a paper-and-pencil questionnaire that was administered in the context of problem gambling prevention classes, aimed at conscripts and held in a Swiss Army recruitment center. The questionnaires were administered between August and December 2019. They were all completed in a 150-seat lecture theatre by groups of conscripts, which varied in size (between 43 and 102 individuals per group). The questionnaires were administered by a prevention team from a problem gambling prevention center, and by military staff who were trained for the task. The questionnaire took about 15 min to complete.

### Questionnaire

The cover page of the questionnaire included information about the purpose, voluntary nature and the ethical standards of the study. Moreover, the cover page included a statement relating to informed consent. After reading this, submission of the completed questionnaire was accepted as consent to participate in the study.

The questionnaire was composed of three sections. The first section was aimed at the whole sample. It included questions about socio-demographic variables (i.e., age, sex, canton of residence, marital status, occupation, highest attained level of education), a definition of the term “gambling”, and two “Yes/No” items inquiring whether the respondents had (a) gambled at least once in their lives and (b) if so, whether they had gambled during the 12 months preceding the study. These two items functioned as filter items. Participants who responded “No” to one or the other filter item were redirected to the third section of the questionnaire.

Those who had reported past-year gambling completed the second section of the questionnaire. The questions investigated gambling frequency, individual net income, the amount of gambling debts if any, offline gambling behavior (i.e., type of gambling, gambling locations, mode of payment, amount spent on gambling), online gambling behaviors (i.e., type of gambling, electronic devices for online gambling, amount spent on gambling), and problem gambling severity through the 9-item Problem Gambling Severity Index (Ferris & Wynne, 2001).

Finally, the third section of the questionnaire included questions intended for lifetime and past-year non-gamblers only. The purpose of this third section was to occupy non-gambling participants while those who gambled completed section two of the questionnaire. The entire questionnaire thus took roughly the same amount of time to complete for all participants and therefore did not reveal to the group which participants had completed section two. The responses received for the third section were irrelevant to the purpose of this study.

### Weighting and Analyses

Data were weighted in order to ensure sample representativeness in terms of age. We performed descriptive analyses to examine gambling-related variables, behaviors and problem gambling prevalence. We then performed crosstab with chi-squared analyses to determine the association of sociodemographic characteristics and gambling measures with problem gambling. To ensure sufficient group size in the contingency categories, the PGSI individual score was dichotomized (No problem and Low risk = Non-problem gambler; Moderate risk and Problem gambling = Problem gambler). All the analyses on gambling variables concerned past-year gambling. We set the level of significance at *p* ≤ 0.05.

## Results

### Gambling Prevalence

Of the 1869 participants, 37.9% reported that they had never gambled in their lives, whereas 62.1% had gambled at least once in their lives. Furthermore, 46.7% reported gambling in the past-year. Finally, 15.4% had gambled at least once in their lives but not in the past year.

### Mode of Access to Gambling and Gamblers’ Income

Almost all past-year gamblers (97.8%) had bet on offline games, whilst 23.6% had gambled online. About 76.4% of the past-year gamblers had bet exclusively on offline games, 21.4% had gambled both on offline games and online, and 2.2% gambled online only. When calculated, for the total sample, these proportions are of 35.7%, 10% and 1% respectively.

With regard to monthly net income, 25.3% had had no income during the 12 months preceding the survey. Six in 10 participants reported a monthly net income not exceeding 4000.- Swiss Francs (CHF), whereas 14% reported an income greater than 4000.- CHF.

### Offline Versus Online Gambling

Table 1 reports offline and online gambling activities during the 12 months preceding the survey. The results show that online gambling yielded more frequent gambling activities than offline gambling. In fact, approximately 25% of the respondents had gambled online at least once per week, compared to 12% of offline gamblers.

**Table 1:** Offline and online past-year gambling

Variables	% Offline (n = 819)	% Online (n = 200)
Gambling frequency		
* Daily*	0.3	1.6
* 2–6 times per week*	4.2	8.2
* Once a week*	7.7	15.8
* 1–3 times per month*	20.3	35.4
* 6–11 times per year*	19.8	15.0
* 1–5 times per year*	47.7	24.0
Type of game		
* Scratch cards*	65.9	9.3
* Roulette*	52.3	28.9
* Sports betting*	36.6	62.3
* Blackjack*	31.4	22.3
* Lottery*	26.2	7.8
* Poker*	24.2	17.5
* Slot machines*	22.7	6.2
* Scratch cards on EGMs*	6.8	–
* Lottery on EGMs*	4.5	0.0
* Horse race betting*	3.0	1.9
* Craps, dice games*	2.8	0.0
* Stock exchange, daily trading*	2.2	9.6
* Games-of-skill betting*	1.8	0.0
* Other*	0.3	2.4
Variety of games		
* 1*	29.6	56.9
* 2*	25.7	27.2
* 3*	15.9	9.2
* 4 or more*	28.7	6.6
Gambling location		
* Vending points*	65.0	–
* Casinos*	64.0	–
* Bars, restaurants*	19.6	–
* Private locations*	18.1	–
* Unofficial backrooms*	4.7	–
* Other locations*	12.2	–
Forms of payment		
* Cash*	93.8	–
* Credit card*	17.2	–
* Pre-paid card*	6.9	–
* E-banking*	1.3	–
* Bank transfer*	1.2	–
* Deposit*	0.6	–
* Postal remittance slips*	0	–
Electronic devices used for gambling		
* Smartphone/Tablet*	–	73.4
* Personal computer*	–	51.2
* Computer at work*	–	3.1
Money spent on gambling		
* None*	1.0	5.6
* 1–50 CHF*	59.8	51.3
* 51–200 CHF*	25.2	35.6
* 201–500 CHF*	11.3	4.4
* 501 CHF or more*	2.7	3.1

As predicted, the type of game played varied according to the form of gambling. Scratch cards dominated amongst offline games (65.9%), closely followed by roulette (52.3%), sports betting (36.6%) and blackjack (31.4%). Lottery (26.2%), poker (24.2%) and slot machines (22.7%), were also popular offline games for this subpopulation. Lottery vending points (65%) and casinos (64%) were the most frequented gambling venues. Bars, restaurants (19.6%) and private locations (18.1%) were also quite often visited. Cash was generally used as a form of payment (93.8%). Approximately one gambler in six used credit cards. Pre-paid cards were used less often (6.9%) and reported use of other forms of payment was negligible.

As far as online gambling is concerned, sports betting was the most popular online game (62.3%). Casino games such as roulette (28.9%), black jack (22.3%) and poker (17.5%) were also relatively popular online games. Moreover, at the time of this study, offline gambling seemed to generate more diversity in types of gambling than did online games. Indeed, the results show that approximately 44% of offline gamblers played three different types of games or more compared to just over 15% of online gamblers. The majority of online gamblers played on smartphones or tablets (73.4%). Around half of them (51.2%) gambled on their personal computer. However, gambling on the computer at work was very infrequent (3.1%).

Globally, no major differences were observed between offline and online forms of gambling concerning the amount money spent in gambling. However, a higher proportion of participants reported that they had not spent money on online gambling, compared to land based gambling. Furthermore, participants reported spending higher amounts (between 201 and 500 CHF) on offline gambling in comparison to online gambling.

#### Problem Gambling Prevalence

According to the PGSI, approximately 80% of the past-year gamblers had no gambling problems or were low-risk gamblers (see Table 2). 17.6% appeared to be moderate-risk gamblers whereas 3.6% were categorized as problem gamblers (i.e., 8.3% and 1.6% of the total sample, respectively).

**Table 2:** Problem gambling prevalence based on the PGSI (n = 833)

	%
Degree of problem gambling severity	
* Non-problem gamblers*	48.7
* Low-risk gamblers*	30.2
* Moderate-risk gamblers*	17.6
* Problem gamblers*	3.6

### Factors Associated with Problem Gambling

#### Occupation, Highest Attained Level of Education, Monthly Net Income

Crosstab analyses revealed no significant association between problem gambling and socio-demographic characteristics of the past-year gamblers (i.e., occupation, highest attained level of education, monthly net income).

#### Mode of Access, Gambling Frequency, Gambling Debts

The associations between mode of access, gambling frequency, and gambling debts with problem gambling are reported in Table 3.

**Table 3:** Associations between mode of access, gambling frequency and gambling debts for past-year gamblers with problem gambling

Variables	Non-problem gamblers (n = 664)	At-risk/problem gamblers (n = 171)	
	%	%	*p*
Mode of access			
* Offline only*	79.5	63.4	**
* Offline and online*	18.6	32.9	
* Online only*	1.8	3.7	
Gambling frequency			
* Once per week or more*	8.8	26.1	***
* One to three times per month*	20.2	22.7	
* One to eleven times per year*	71.0	51.2	
Gambling debts			
* No*	99.3	99.1	*ns*
* Yes*	0.7	0.9	

*ns* non-significant; *p < 0.05;**p < 0.01; ***p < 0.001

Problem gambling was significantly associated with the mode of access to gambling (X^2^ (1.63, N = 833) = 20.081; *p* < 0.005). Specifically, online only and the mixed offline and online modes of access were more specific of at risk/problem gamblers than of non-problem gamblers. Indeed, approximately a third of at-risk/problem gamblers played both offline and online, compared to 18.6% of non-problem gamblers. With regard to the online only mode of access, the proportion of at-risk/problem gamblers was twice that of non-problem gamblers.

Problem gambling was also significantly associated with gambling frequency (X^2^ (2.00, N = 829) = 41.594; *p* < 0.001). At-risk/problem gamblers were approximately three times more likely to report gambling at least once a week, when compared to non-problem gamblers.

No significant association was found between gambling debts and the presence of at risk/problem gambling.

#### Gambling Behaviors

We performed crosstab analyses to examine the associations between gambling behaviors and problem gambling. The analyses were performed separately for offline and online gambling behaviors. The results concerning the associations between offline behaviors and problem gambling are reported in Table 4. No significant associations emerged between online-gambling behaviors and problem gambling, and are therefore not displayed in the table.

**Table 4:** Association of offline gambling behaviors with problem gambling

Variables	Non-problem gamblers (n = 644)	At-risk/problem gamblers (n = 160)	
	%	%	*p*
Number of different offline games played			
* 1*	31.3	21.9	**
* 2*	28.1	16.1	
* 3*	14.5	22.8	
* 4 or more*	26.2	39.2	
Gambling locations			
* Lottery vending points*	71.7	73.3	*ns*
* Casinos*	61.0	78.8	***
* Bars and restaurants*	20.2	18.5	*ns*
* Private locations*	18.0	19.7	*ns*
* Unofficial backrooms*	3.3	9.8	*
Forms of payment			
* Cash*	92.8	98.4	**
* Credit card*	15.4	24.4	*
* Pre-paid card*	5.9	10.6	*ns*
* E-banking*	0.9	2.5	*ns*
* Bank transfer*	1.1	1.9	*ns*
* Deposit*	0.3	1.9	*ns*
* Postal remittance slip*	0.0	0.0	*ns*
Money spent on offline gambling			
* None*	3.4	4.3	***
* 1–50 CHF*	80.3	46.1	
* 51–200 CHF*	12.0	31.5	
201 *CHF or more*	4.3	18.2	

*ns* non-significant; *p < 0.05;**p < 0.01; ***p < 0.001

Problem gambling was associated with several offline-gambling behaviors. Firstly, it was associated with the variety of games used (X^2^ (2.99, N = 804) = 25.024; *p* < 0.005). Indeed, problem gamblers bet less often on one or two different types of games, and more often on three or more different types of games, in comparison to non-problem gamblers.

Particularly, problem gambling was related to two specific offline gambling locations, namely casinos (X^2^ (1, N = 804) = 18.462; *p* < 0.005) and backrooms/unofficial locations (X^2^ (1, N = 804) = 12.446; *p* < 0.02). In both locations, problem gamblers were more represented than non-problem gamblers. No differences were found for the other gambling locations.

The form of payment too was significantly related to problem gambling, specifically the use of cash (X^2^ (1, N = 804) = 7.479; *p* < 0.005) and of credit cards (X^2^ (1, N = 804) = 7.480; *p* < 0.05). Problem gamblers used these two forms of payment more often than non-problem gamblers. No significant differences between problem gamblers and non-problem gamblers were observed for the other forms of payment considered.

Finally, crosstab analyses yielded a significant association between the amount of money spent on gambling and the presence of problem gambling behaviors (X^2^ (2.99, N = 781) = 85.878; *p* < 0.001). Unsurprisingly, problem gamblers reported higher levels of spending on gambling, compared to non-problem gamblers.

## Discussion

The first aim of the study was to examine offline and online gambling behaviors in emerging-adult males in the French-Speaking region of Switzerland. We found that approximately 47% of the respondents had gambled in the 12 months preceding the inquiry, which is close to the finding recently reported elsewhere (Wardle & McManus, 2021). We also determined that more than three in four past-year gamblers had bet exclusively offline, whereas one in four had also gambled online. Thus, despite the ongoing expansion of online gambling and associated ease of access, offline gambling remains more popular amongst these youths (Conolly et al., 2018). However, when compared to the previous gambling prevalence findings on this same population of Swiss emerging-adult males (56%; Tomei et al., 2015), the present results suggest that gambling activities in this population have slightly decreased over the past seven years. On the other hand, online gambling activities seem to have increased. Indeed, we found that approximately 24% of past-year gamblers had bet online, compared to previous reports (16%; Tomei et al., 2015). This opposing trend of a decrease in gambling activity in general, and an increase in online gambling has also been observed elsewhere (Conolly et al., 2018).

Concerning electronic devices to access online gambling, personal computers were used by approximately 50% of online gamblers, whereas smartphones or tablets were used by close to 75% of them. Particular attention should be paid to the use of mobile devices in gambling as they might contribute to the development and maintenance of problem gambling (James et al., 2017).

For a better grasp of gambling behaviors in our population of interest, we analyzed both offline and online gambling. The comparison of these forms of access yielded several noteworthy differences. For instance, emerging adult males appear to gamble more frequently online than they do offline. One possible explanation is that higher gambling frequency may be related to the fact that over 70% of online gamblers played their smartphone or tablet, making gambling more accessible to them than offline games. On the other hand, whilst offline gambling was performed on a variety of games (approximately 29% of the gamblers played four or more different games), online gambling was limited to one or two different games. Sports betting was the major online gambling activity; a finding that has also been observed elsewhere (Wardle & McManus, 2021). The popularity of online betting should raise concerns as some of its structural characteristics, such as live betting and micro-event betting, may generate problem-gambling behaviors (Parke & Parke, 2019). Sports betting is quite popular offline too. However, scratch cards and casino games such as roulette and blackjack are more prevalent offline games. Indeed, consistent with offline game preferences, lottery vending points and casinos were the most visited gambling venues. Approximately one in five gamblers waged money in bars and restaurants. However, they also gambled in private locations at a similar rate. Private gambling has been shown to have negative outcomes in terms of problem gambling and substance use (Studer et al., 2016). Moreover, gambling in private locations could present a major concern in terms of early detection and prevention efforts. In fact, whilst gambling activities in the casinos and establishments with video lottery terminals must take place under the supervision of staff and mangers trained as part of a *responsible gambling* program, gambling in private locations eludes this scrutiny. Additionally, the expansion of online gambling may move away from this form of surveillance even more.

With respect to expenditure, the amount of money spent on gambling was quite analogous between the offline and online forms of access, except for spending between 201 and 550 CHF, which appeared to occur more frequently in offline gambling venues. One difference found between the two forms of gambling was that approximately 6% of online gamblers reported no spending on gambling. This may be due to the opportunity given to new customers to gamble for free, on particular websites, for a limited period of time.

The second aim of the study was to measure problem gambling severity rates amongst these emerging-adult males. Responses to the PGSI (Ferris & Wynne, 2001) yielded that 3.6% of past-year gamblers met the criteria of problem gambling, which corresponds to 1.6% of the population. Hence, these prevalence rates are two to three times smaller than those reported in other jurisdictions (Scholes-Balog et al., 2014; Wardle & McManus, 2021). However, the problem gambling prevalence rates yielded in the present study are four times larger than those observed in the general population (Billieux et al., 2016).

The third and final aim of the study was to identify the factors associated with problem gambling. We found no association between problem gambling and the socio-demographic variables measured (i.e., occupation, highest attained level of education, and monthly net income). However, problem gambling was associated with the mode of access to gambling and gambling frequency. Specifically, the proportion of at-risk/problem gamblers was approximately twice that of non-problem gamblers among online-only gamblers. This result confirms previous findings that have shown higher rates of problem gamblers among online users in young populations (Chóliz, 2016; Hing et al., 2015; McCormack et al., 2014; Petry & Weinstock, 2007; Potenza et al., 2011; Wijesingha et al., 2017). Unsurprisingly, at-risk/problem gamblers were also more frequent gamblers.

We also examined the relationship between gambling behaviors and problem gambling separately for offline and online gambling behaviors. As regards to offline gambling behaviors, the results yielded a significant relationship between the variety of different games played and problem gambling. They showed that at-risk/problem gamblers had wagered on a greater variety of games than their non-problem counterparts. This trend is in line with previous reports (Holtgraves, 2009; Kessler et al., 2008; Tomei et al., 2015). The results also showed that problem gambling is associated with wagering specifically in casinos and in unofficial gambling backrooms. Other locations such as lottery vending points, bars, restaurants and private locations did not relate to problem gambling. This is explainable by the fact that casinos and unofficial backrooms are more suitable for intense gambling activities. Indeed, differently from lottery vending points, bars, restaurants and private locations such as private residences, casinos and gambling unofficial backrooms are conceived with the sole purpose of allowing prolonged and intense gambling activities to take place. Unsurprisingly, the results also showed that at-risk/problem gamblers had wagered greater amounts of money and had a greater propensity to bet using cash and credit cards than non-problem gamblers.

A few limitations should be considered when interpreting the results. Firstly, the sample had a limited age span, since it included only young men aged 18–22 years. As gambling activities have been shown to peak between 22 and 30 years of age (Welte et al., 2011), the inclusion of older participants in the analysis may have resulted in higher prevalence rates. Moreover, because of time constraints in administering the questionnaire, several possible explanatory factors for problem gambling, pertinent to this sub-population were left out. We therefore did not examine factors related to gambling onset, social environment associated with gambling (i.e., family members, peers), and potential comorbidities. Findings related to these factors bring precious information, which can be used for prevention purposes. To overcome these limitations, future research should include a larger sample with older age groups. In addition, future waves of this survey should include a broader array of items to provide insightful information on the possible explanatory factors for problem gambling within this population. Furthermore, to develop a fuller picture of gambling activities and problem gambling in emerging adults in Switzerland, investigation should include female participants too. Indeed, even though young men gamble more and are at higher risk of problem gambling than their female counterparts, in the light of the development of online gambling, greater efforts should be deployed to include women in gambling studies.

To conclude, the present study shows that as the new Swiss Act on Gambling has entered into force, less than 50% of emerging-adult males in French-speaking Switzerland gamble. Indeed, this rate has even decreased over recent years. Problem gambling rates appear to be lower than the ones observed for the same sub-population in other jurisdictions. However, we observed that online gambling is on the rise. The findings also show that online gambling is associated with problem gambling. Since the online gambling market is expanding in Switzerland, it is crucial that gambling activities in this population, particularly online gambling, are monitored and assessed on a regular basis. In fact, public health stakeholders have begun a consultation in order to set up a monitoring process for gambling activities in Switzerland. It is equally important to pursue prevention efforts in terrestrial gambling venues, namely with emerging adults in casinos. Finally, research should be conducted to gain a better understanding of gambling activities in private locations, particularly in unofficial backrooms.
